# Burnout and Psychological Safety: A Questionnaire Study of Medical Students

**DOI:** 10.1177/23821205251359380

**Published:** 2025-07-20

**Authors:** Aziz Bitar, Jens Boman, Emelie Kristoffersson

**Affiliations:** 1 Department of Clinical Science, Professional Development, 8075 Umeå University, Umeå, Sweden

**Keywords:** burnout, psychological safety, burnout assessment tool, questionnaire, Amy Edmondson’s team psychological safety survey, undergraduate medical students

## Abstract

**Background:**

Psychological safety—defined as the perception that a group environment is safe for interpersonal risk-taking—could influence the development of burnout among medical students. This study, therefore, sought to investigate the prevalence of burnout, the level of psychological safety, and their association in this population.

**Methods:**

We surveyed undergraduate medical students (*n* = 944) enrolled in semesters 2 to 10 at a Swedish medical school using a questionnaire containing the Burnout Assessment Tool (BAT-12), Amy Edmondson's Team Psychological Safety Survey, and socio-demographic questions. Data were analyzed using descriptive statistics, *t*-tests, one-way ANOVA, Pearson's correlation, and hierarchical regression analysis.

**Results:**

A total of 457 medical students (62.6% women) completed the questionnaire (response rate: 48%). Of the participants, 51.4% scored above the cut-off for burnout risk, and among these, 25.6% were at very high risk. Women scored significantly higher on burnout and lower on psychological safety than men. A significant negative association was found between psychological safety and burnout, with psychological safety accounting for an additional 14.3% of the variance in burnout scores.

**Conclusions:**

Our findings suggest that more than half of the surveyed medical students are at risk for burnout and that lower levels of psychological safety are associated with higher levels of burnout. Furthermore, women appear to be at higher risk for burnout and perceive medical school as less psychologically safe than male students. While longitudinal studies are needed to assess the causality between low psychological safety and burnout, our results provide impetus for developing interventions to prevent burnout and addressing an eventual lack of psychological safety in medical school.

## Background

Burnout—traditionally regarded as a marker of professional distress—seems to originate as early as medical school.^1–5^ Characterized by exhaustion, mental distance, and both cognitive and emotional impairment,^6^ burnout exerts a negative influence on medical students’ well-being, often persists throughout their careers,^5^ and is associated with anxiety, depression,^7^ and suicidal ideation.^8^ A multinational meta-analysis estimated the prevalence of burnout among medical students at 44.2%,^4^ indicating a crisis in the mental health of medical students. Moreover, some studies have reported higher rates of burnout among female medical students.^9^

Both individual and curricular factors,^2^ as well as repeated mistreatment by faculty and residents,^10^ have been associated with burnout among medical students. Notably, medical students tend to enter their studies with lower levels of burnout compared to other student populations.^2,11^ However, by their second year, they exhibit higher burnout rates than the general population,^12^ suggesting that medical education itself may contribute to the development of burnout. Still, little is known about the factors within the educational milieu that may influence the risk for burnout,^13^ highlighting the need to examine factors such as psychological safety in the learning environment.

Drawing on the work of Amy Edmondson, psychological safety refers to an interpersonal climate of respect and trust within a group—such as a student group or workplace—where individuals feel safe to take interpersonal risks and show vulnerability, for example, admitting mistakes and asking for help without fear of negative consequences.^14^ Studies in health professions education have shown that psychologically safe clinical learning environments enable students to ask questions and acknowledge gaps in their knowledge without concern for their image.^15,16^ Psychologically unsafe learning environments, in contrast, can leave students feeling embarrassed, dehumanized, isolated, and lacking in confidence.^17^

Unfortunately, studies indicate that many necessary components facilitating psychologically safe learning environments are lacking for medical students worldwide.^17^ The high stakes associated with medical errors have contributed to a culture of perfectionism and competition, where students’ performance is under constant scrutiny.^18^ As a result, students often report a fear of making mistakes, coupled with pressure to conceal uncertainty^19^ and a reluctance to seek help for mental illness.^19,20^ Moreover, many medical students are subjected to mistreatment from supervisors,^10,21^ behaviors that negatively impact the learning environment.^22^ These findings raise the question of how psychological safety—or the lack thereof—might impact medical students’ well-being and their risk of burnout.

While burnout among medical students—and its higher prevalence among women—has been well documented, less is known about its potential correlation with psychological safety. Recent studies from practicing nurses and physicians suggest that lower levels of psychological safety are associated with higher burnout, particularly in the form of work exhaustion among trainees.^23,24^ However, this relationship has not yet been explored among medical students. Gaining a better understanding of this possible association is needed to inform curricular reforms aimed at improving medical students’ well-being. Therefore, we sought to investigate the prevalence of burnout, the level of psychological safety, and their association among undergraduate medical students. Additionally, we examined differences in psychological safety and burnout across subgroups of students based on gender, age, and attending semester.

## Methods

### Study Design and Setting

As part of a broader project on students’ well-being at Umeå University, Sweden, we conducted a cross-sectional study to investigate the prevalence of burnout, the level of psychological safety, and their correlation among medical students.

In Sweden, undergraduate medical education is undergoing a transition from a 5.5-year program followed by an 18- to 24-month internship to a 6-year program that grants students a medical license upon graduation. Semesters 1 to 5 are pre-clinical, while 6 to 11/12 are clinical. At the time of data collection, students in semesters 2 to 4 were enrolled in the newly implemented 6-year program (launched in autumn 2021), while students in semesters 5 to 10 were part of the former 5.5-year program. Although the new program includes an additional clinical semester (semester 12), no participants had yet reached that stage, and clinical exposure up to semester 10 was comparable across both programs.

The reporting of this study conforms to the STROBE (Strengthening the Reporting of Observational Studies in Epidemiology) guidelines for cross-sectional studies^25^ (see Supplemental Material 1: STROBE checklist).

### Measures

Using Microsoft Forms, we developed a web-based questionnaire that included sociodemographic items, assessing students’ semester of study, age group, and gender, as well as validated scales assessing burnout and psychological safety.

#### Burnout Assessment Tool (BAT-12)

Burnout was measured using the Burnout Assessment Tool (BAT-12), a shortened version of the full 23-item BAT, consisting of 12 items.^26^ The BAT-12 comprises 4 subscales: *exhaustion* (ie, extreme tiredness), *mental distance* (ie, psychological withdrawal), and *cognitive-* and *emotional impairment* (ie, difficulties in regulating cognitive and emotional processes, respectively).^6^ Participants rate each item on a 5-point Likert scale, and mean scores are then calculated for each participant. Clinically validated cut-off scores for BAT-12 categorize participants into 3 subgroups or risk groups: green (< 2.54), indicating not at risk; orange (2.54-2.96), suggesting being at risk; and red (> 2.96), indicating potential burnout.^6^ The Swedish version of BAT has been validated.^27^ To adapt the BAT to the educational context of this study, the word “work” in the original items was replaced with “school,” following the validated student version of the BAT.^28^ For example, the item “*After a day at work, I find it hard to recover my energy*” was modified to “*After a day at school, I find it hard to recover my energy.*” In the current study, the BAT-12 demonstrated good internal consistency, with a Cronbach's alpha coefficient of .87.

#### Team Psychological Safety Survey

Psychological safety was assessed using Amy Edmondson's Team Psychological Safety Survey.^14^ The survey consists of 7 items, for example, “If you make a mistake in this team, it is often held against you” and “It is difficult to ask other members in this team for help,” rated on a 7-point Likert scale. The total score thus ranges from 7 to 49. There are no established subgroups or cut-off scores for the psychological safety survey. Therefore, consistent with previous studies in medical education and healthcare settings, an index score was calculated by averaging responses across the 7 items, yielding a scale from 1 to 7, where higher scores reflect greater perceived psychological safety.^24,29^ Permission to use the Team Psychological Safety Survey was obtained from Amy Edmondson. The survey was professionally translated from English to Swedish and then back-translated to ensure accuracy. The final version was reviewed and approved by Sander Hoeken and Amy Edmondson. To adapt the survey to the educational context of this study, the term “team” in the original scale was replaced with “student group,” reflecting the peer-based learning environment in which medical students primarily engage at our institution. The Team Psychological Safety Survey has been widely used in healthcare education and clinical settings. It has demonstrated good internal consistency among European medical students, nurses, surgical trainees, and faculty.^23,24,29^ In the present study, the Swedish-translated version yielded a Cronbach's alpha of .80, supporting its reliability in our target population.

### Data Collection

In spring 2023, we invited all medical students registered in semesters 2 through 10 at Umeå University to participate in the study via the university's learning management system, Canvas. No a priori power analysis was conducted. However, nearly all eligible medical students at Umeå University (*n* = 944) were invited to participate in the study, and 457 students responded (response rate: 48%). The invitation included information about the study's purpose, emphasized that participation was anonymous and voluntary, and provided a link to the questionnaire. To encourage participation, we sent 2 reminders to complete the questionnaire. Because participation was anonymous and voluntary, no data were collected on the reasons for non-participation.

### Statistical Analyses

We processed the collected data in Microsoft Excel and subsequently imported it into IBM SPSS Statistics 28 for analysis. All questionnaires were complete, with no missing values across any variables.

The normality of the distributions for the variables was assessed using Q–Q plots and descriptive statistics, including skewness and kurtosis. No substantial deviations from normality or extreme values were observed. Burnout and psychological safety scores were calculated for each participant, and comparisons were made across demographic groups.

Separate one-way ANOVA analyses were conducted to compare burnout and psychological safety scores across age groups and semester groups. Due to small sample sizes in the older age groups, participants aged 35 and above were recoded into a single group labeled > 35 for statistical purposes. Independent samples *t*-tests were used to compare burnout and psychological safety scores between male and female students.

Pearson's correlation analysis was performed to investigate the relationship between psychological safety and burnout scores. To further investigate this relationship, a hierarchical regression analysis was conducted. In Step 1, gender (coded as 1 =  woman, 2  =  man) was entered into the model as a control variable. In Step 2, psychological safety was added to the model to assess its additional contribution to explaining variance in burnout scores.

Six participants identified their gender as “other.” Due to the small sample size, this group was excluded from group comparisons and regression analyses but included in descriptive statistics, burnout subgroup distributions, and correlation analyses.

### Ethical Considerations

Participants received information about the study and provided informed consent to participate by voluntarily completing the questionnaire. Participation was optional, the questionnaire was anonymous, and no personal data were collected. Consequently, following The Swedish Act (2003:460) concerning the ethical review of research involving humans, the study was deemed exempt from the requirement for approval by the Swedish Ethical Review Authority.

## Results

Of the 944 medical students invited to participate, 457 responded, resulting in a response rate of 48%. Of the participants, 62.6% identified as women, 36.1% as men, and 1.3% as others. The largest age group was those  <  25 years, representing 57.2% of the participants.

Demographic characteristics, including gender, semester of study, and age group, are presented in Table 1.

**Table 1.** Demographic Characteristics of the Participants and Mean Scores of Psychological Safety and Burnout.

	Number of participants (%)	Psychological safety (± SD)	Burnout (± SD)
Overall	457 (100.0%)	5.39 ± 1.02	2.61 ± 0.61
Attending semester			
2	52 (11.4%)	4.87 ± 1.17	2.56 ± 0.60
3	53 (11.6%)	5.18 ± 1.01	2.64 ± 0.67
4	50 (10.9%)	5.18 ± 1.01	2.57 ± 0.71
5	50 (10.9%)	5.02 ± 1.03	2.69 ± 0.60
6	51 (11.2%)	5.02 ± 1.03	2.55 ± 0.56
7	44 (9.6%)	4.83 ± 1.07	2.64 ± 0.59
8	50 (10.9%)	5.38 ± 1.05	2.61 ± 0.56
9	54 (11.8%)	4.93 ± 1.02	2.79 ± 0.72
10	53 (11.6%)	5.31 ± 0.83	2.48 ± 0.46
Age			
≤ 24	262 (57.2%)	5.07 ± 0.95	2.63 ± 0.58
25-29	129 (28.4%)	5.17 ± 1.03	2.60 ± 0.68
30-34	46 (10.0%)	5.12 ± 1.13	2.61 ± 0.57
≥ 35	20 (4.4%)	4.94 ± 1.51	2.43 ± 0.71
Gender			
Female	286 (62.6%)	5.00 ± 1.01	2.69 ± 0.56
Male	165 (36.1%)	5.30 ± 1.00	2.47 ± 0.67
Other	6 (1.3%)	4.24 ± 0.87	2.92 ± 0.59

*Note.* SD, standard deviation. Psychological safety index scores range from 1 (low) to 7 (high). Burnout score interpretation is based on validated cut-off ranges:  < 2.54 (no risk),  2.54–2.96 (at risk),  > 2.96 (very high risk).

### Categorization of Burnout Based on Cut-Off Scores

Of all participants, 51.4% scored above the clinically validated cut-off scores, indicating they were at risk for experiencing burnout. Upon further classification based on the scoring guidelines, 48.6% of participants were *not at risk*, 25.8% were *at risk*, and 25.6% were *at very high risk* for burnout.

The distribution of participants across the burnout risk subgroups is presented in Table 2.

**Table 2.** The Distribution of Participants Between Different Burnout Subgroups.

Subgroup	Number of participants	(%)
Green (no risk)	222	48.6
Orange (at risk)	118	25.8
Red (very high risk)	117	25.6

*Note.* Classification using a traffic light system analogy: green indicates no risk of burnout, orange indicates at risk for burnout, and red indicates very high risk for burnout.

### Categorization of Psychological Safety Levels

The average psychological safety score among all participants was 5.39, suggesting a moderately high level of perceived psychological safety.

### Comparison of Burnout Scores by Demographic Characteristics

The overall mean burnout score among participants was 2.61, placing the average student in the *orange* category, indicating they were at risk for burnout. An independent samples *t*-test indicated that women had significantly higher burnout scores than men, *t*(449) = 5.087, *P* < .001, with a small effect size (*d* = 0.38). A one-way ANOVA showed that attending semester did not significantly affect burnout scores, *F*(8, 448) = 1.15, *P* = .331. Similarly, a one-way ANOVA showed that burnout scores did not differ significantly across age groups, *F*(3, 453) = 0.72, *P* = .543.

Participants’ mean burnout scores are presented in Table 1.

### Comparison of Psychological Safety by Demographic Characteristics

An independent samples *t*-test showed that women reported significantly lower psychological safety scores than men, *t*(449) = –3.05, *P* = *<* .001, with a small effect size (*d* = 0.30). A one-way ANOVA revealed no significant effect of attending semester on psychological safety scores, *F*(8, 448) = 1.93, *P* = .053. Similarly, a one-way ANOVA revealed no significant difference in psychological safety scores across age groups, *F*(3453) = 0.46, *P* = .710.

The mean psychological safety scores for participants by demographic subgroup are presented in Table 1.

### Correlation Between Psychological Safety and Burnout

Correlation analysis revealed a significant negative association between psychological safety and burnout scores (*r* = −.400, *P* < .001), indicating that higher levels of psychological safety were associated with lower levels of burnout.

**Figure 1.**
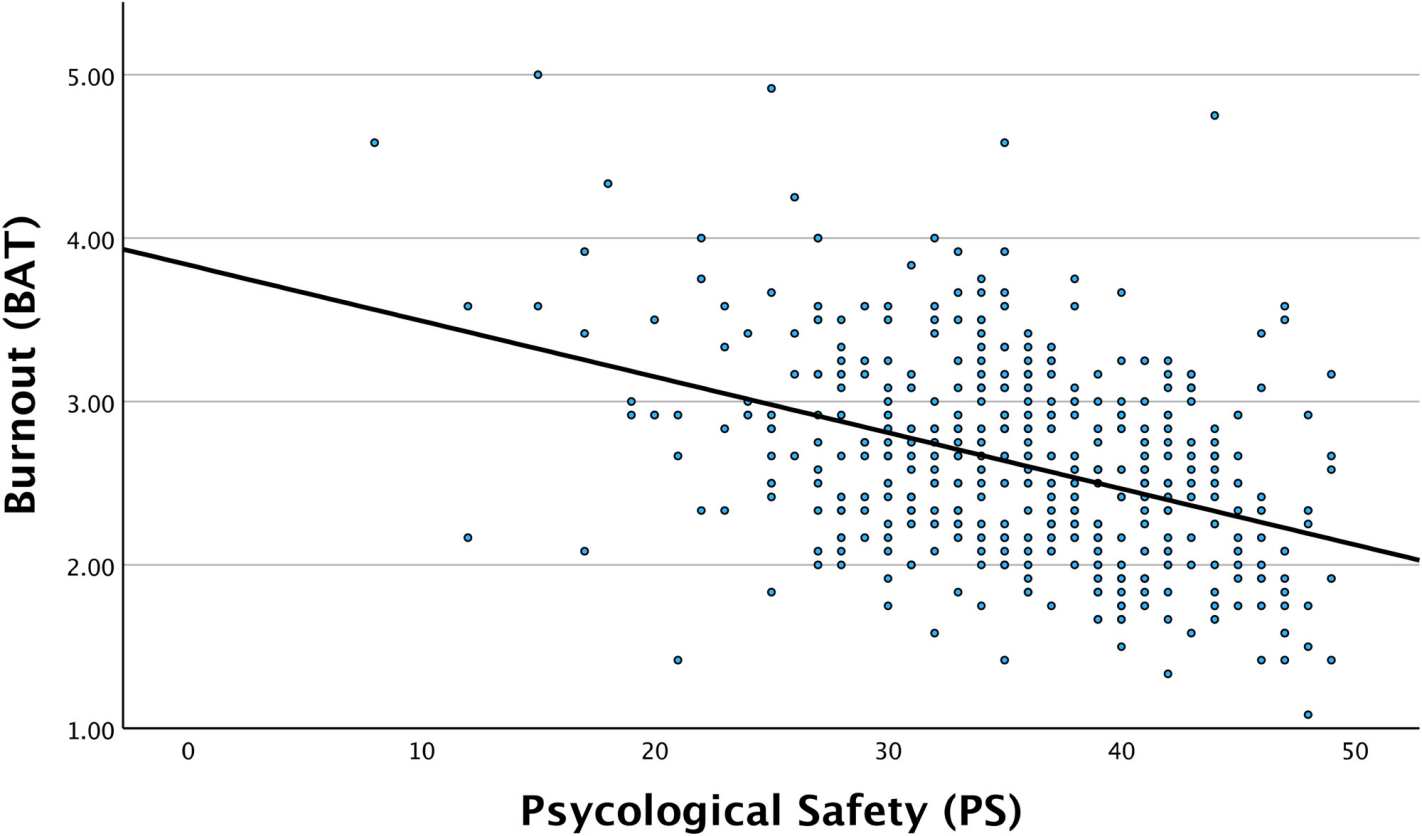
Correlation Between Psychological Safety and Burnout Scores Among Medical Students (*n* = 457). Pearson Correlation Coefficient (ρ = −0.400; *P*-value = < .001).

The correlation analysis between psychological safety and burnout scores is illustrated in Figure 1.

### Hierarchical Regression on Burnout

In Step 1, gender was entered into the model as a control variable, and it was found to be significantly associated with burnout, accounting for 3.2% of the variance in burnout scores, *F*(1, 449) = 14.88, *P* < .001.

In Step 2, psychological safety was added to the model. The inclusion of psychological safety significantly improved the model fit, explaining an additional 14.3% of the variance in burnout scores, Δ*R*² = 0.143, *F*(2, 448) = 77.65, *P* < .001. The final model, which included both gender and psychological safety, accounted for 17.5% of the total variance in burnout scores (Δ*R*² = 0.175).

In the final model, both gender (*β* = −0.125, *P* = .004) and psychological safety (*β* = −0.382, *P* < .001) were significantly associated with burnout scores.

The summarized results from the hierarchical regression analysis are presented in Table 3.

**Table 3.** Hierarchical Regression with Burnout as Outcome Among Medical Students.

Step	Variables	*R*	*R*²	Δ*R*²	*F* Change	*P*
1	Gender	0.179	0.032	0.032	14.879	<.001
2	Gender, Psychological Safety	0.418	0.175	0.143	77.650	<.001

*Note.* The dependent variable is burnout.

Δ*R*² represents the change in explained variance between steps.

## Discussion

To our knowledge, this is the first study to report the prevalence of burnout, the level of psychological safety, and their association among undergraduate medical students. Of all participants, 51.4% scored above the clinically validated cut-off scores for burnout, indicating they were at risk of experiencing burnout. Of these, 25.6% were at very high risk. Notably, women scored significantly higher than men. Furthermore, students had an average psychological safety score of 5.4, with women scoring significantly lower than men. Neither attending semester nor age group had a significant impact on burnout or psychological safety scores. Most importantly, our findings revealed a negative association between psychological safety and burnout scores. Even after controlling for gender, psychological safety remained a significant protective factor against burnout. Thus, this study contributes to the ongoing discussion about the factors that contribute to and sustain poor mental health among medical students.

Our findings suggest that the average Swedish medical student is at risk for burnout, with 25.6% classified as being at high risk—prevalences comparable to those reported among medical students in other countries.^4^ Furthermore, in line with some previous studies,^9^ women in our study scored significantly higher on burnout than men. This gender difference could be partially explained by women being more exposed to discrimination and harassment,^30,31^ as recurrent mistreatment has been shown to be associated with burnout.^10^ Another contributing factor to this finding could be related to women perceiving the educational environment as less psychologically safe—a point we will elaborate on below. In contrast, we found no significant effect of the semester or age group on burnout scores, indicating that students experience burnout early in their education and experience little to no relief as they progress through their studies. The high levels of burnout observed in both our study and in previous research^4^ are concerning—not only for the well-being of individual students and physicians but also for the maintenance of competence in healthcare.

Our participants had an average psychological safety score of 5.4, which is slightly higher than the average score of 4.9 reported in a large European study of medical students,^29^ thus suggesting a relatively high level of perceived psychological safety. However, our data suggest that female students perceive the study climate as less psychologically safe than their male peers. This gender difference may reflect the persistence of outdated masculine ideals in medicine, alongside enduring gender stereotypes that shape perceptions of who is considered a legitimate future physician. The physician ideal remains largely male-coded—emphasizing autonomy, decisiveness, emotional detachment, and self-sufficiency.^32–35^ Accordingly, research shows that men are more readily perceived as fitting this role.^36^ In contrast, women often feel compelled to prove their competence and, as a result, may struggle more with expressing uncertainty.^36^ The lower levels of psychological safety reported by our female participants may, therefore, reflect the tension they experience in trying to conform to a physician's ideal that discourages vulnerability.

Moreover, the results showed a significant association between low psychological safety and high burnout scores, findings supported by recent research. In the previously mentioned large European study of medical students, lower psychological safety was significantly linked with higher levels of burnout, particularly among students who reported experiences of mistreatment or a lack of supportive relationships with faculty members.^29^ Similarly, a recent study among surgical trainees reported that lower psychological safety was associated with increased emotional exhaustion.^24^ These consistent findings across different groups of learners support the notion that a psychologically unsafe environment is tied to a heightened risk of burnout. The reason that low psychological safety leads to increased burnout could be that a study and work environment where neither mistakes, anxiety, insecurity, nor questions are tolerated likely creates a high level of stress. Our cross-sectional design does not allow for determining a cause-and-effect relationship between psychological safety and burnout. However, given that previous studies also show that students start medical school with mental health profiles resembling those of non-medical student peers,^2,11^ which then deteriorates,^12^ our study strengthens the theory that the education itself impairs students’ psychological well-being—and here low psychological safety could be a mediating factor.

Although our participants, on average, scored relatively high on psychological safety, it is alarming that some students perceive medical school as an environment where disclosing uncertainty and sharing experiences of making mistakes feels unsafe. This finding is problematic for several reasons—not only because of its potential association with burnout. First, errors and not knowing are ubiquitous in medicine.^37^ Second, exposing insecurity, asking questions, and raising opinions are critical to medical students’ learning process.^16^ Third, admitting uncertainty and asking questions, we believe, is a prerequisite for reducing the risk of medical errors.

## Medical Education Implications

Our findings emphasize the importance of early interventions to address medical student burnout. Efforts such as conversion to pass-fail grading systems and participation in self-development groups have shown promising results in mitigating burnout.^38^ However, considering the potential link between burnout and low psychological safety, it is also important to explore whether interventions to enhance psychological safety can also prevent and alleviate burnout. A previous study found that medical students’ perception of whether the study climate was psychologically safe or unsafe was primarily based on their observations of how clinical supervisors behaved and interacted with students.^17^ Consequently, future intervention studies should focus on supporting supervisors in adopting leadership styles that promote psychological safety. Amy Edmondson has described 3 leadership characteristics that build the relationships of trust and respect fundamental to psychological safety.^39^ First, set the stage by clearly defining expectations and destigmatizing failure. Second, invite participation by encouraging input and demonstrating humility and openness to change. Third, respond productively by offering support and expressing appreciation. Promoting such leadership styles may also help challenge the obsolete and health-threatening norms on self-sufficiency, emotional detachment, and unwavering decisiveness that continue to dominate the culture of medicine.

## Limitations

Our study has limitations that should be considered when interpreting the findings. First, the respondents may not fully represent the broader population of medical students at Umeå University. For example, it is plausible that students experiencing higher levels of burnout and lower psychological safety were more inclined to participate, potentially leading to selection bias. Second, the cross-sectional design does not allow for determining a cause-and-effect relationship between psychological safety and burnout. Third, while this study utilized the BAT-12 to measure burnout, most previous studies^4^ have employed the Maslach Burnout Inventory Survey (MBI),^40^ making direct comparisons difficult. However, our use of the BAT can also be viewed as a strength, as it provides a more comprehensive conceptualization of burnout. Continued research and validation of the BAT scale will determine whether this tool can evolve into the new gold standard for assessing burnout. Fourth, that we did not conduct a formal a priori power analysis may have limited our ability to detect small effect sizes. Finally, the generalizability of our results may be limited, as the sample was restricted to a single medical school. Nevertheless, within the bounds of these limitations, our study offers the first insights into the association between psychological safety and burnout among undergraduate medical students—results that are likely also of relevance to other health professional students. Furthermore, the study had a reasonably high response rate, strengthening the reliability of the results.

## Conclusions

Our results indicate that more than half of all medical students are at risk of burnout and that a psychologically unsafe educational environment is associated with higher levels of burnout. Additionally, women appear to be at a greater risk for burnout and perceive the learning environment as less psychologically safe than their male co-students. These findings underscore the importance of creating a psychologically safe educational environment where students feel comfortable asking questions, expressing uncertainty, and acknowledging mistakes. Future longitudinal intervention studies should investigate approaches for training supervisors in leadership styles that promote psychologically safe medical school climates by setting clear expectations, encouraging students to ask questions, and creating a focus on learning and constructive feedback. These interventions could enhance medical students’ sense of psychological safety and, in turn, reduce the prevalence of burnout. Equipping supervisors with these leadership skills could also play a pivotal role in changing health-threatening norms and obsolete ideals—such as emotional detachment, self-sufficiency, and decisiveness—that still prevail in medicine.

## Supplemental Material

sj-docx-1-mde-10.1177_23821205251359380 - Supplemental material for Burnout and Psychological Safety: A Questionnaire Study of Medical Students Supplemental material, sj-docx-1-mde-10.1177_23821205251359380 for Burnout and Psychological Safety: A Questionnaire Study of Medical Students by Aziz Bitar, Jens Boman and Emelie Kristoffersson in Journal of Medical Education and Curricular Development

sj-pdf-2-mde-10.1177_23821205251359380 - Supplemental material for Burnout and Psychological Safety: A Questionnaire Study of Medical Students Supplemental material, sj-pdf-2-mde-10.1177_23821205251359380 for Burnout and Psychological Safety: A Questionnaire Study of Medical Students by Aziz Bitar, Jens Boman and Emelie Kristoffersson in Journal of Medical Education and Curricular Development

## List of Abbreviations

BAT-12: burnout assessment tool
