# Supplementary material for: Burnout and Psychological Safety: A Questionnaire Study of Medical Students
Source: J Med Educ Curric Dev. 2025 Jul 20;12:23821205251359380. doi: 10.1177/23821205251359380 (PMC12277557; doi:10.1177/23821205251359380)
Supplement: sj-pdf-2-mde-10.1177_23821205251359380 - Supplemental material for Burnout and Psychological Safety: A Questionnaire Study of Medical Students [file sj-pdf-2-mde-10.1177_23821205251359380.pdf]

Bakgrundsfrågor

Ditt kön

- ◯ Kvinna
- ◯ Man
- ◯ Den indelningen passar inte mig

Din ålder

- ◯ -24
- ◯ 25-29
- ◯ 30-34
- ◯ 35-39
- ◯ 40-44

Vilken termin går du?

- ◯ T2
- ◯ T3
- ◯ T4
- ◯ T5
- ◯ T6
- ◯ T7
- ◯ T8
- ◯ T9
- ◯ T10

Din känsla av utmattning

Följande påståenden handlar om din situation som student och hur du upplever den. Vänligen ange hur ofta varje påstående gäller för dig.

När jag är i skolan känner jag mig psykiskt utmattad.

- ◯ Aldrig
- ◯ Sällan
- ◯ Ibland
- ◯ Ofta
- ◯ Alltid

Det är svårt för mig att återhämta mig efter en dag i skolan.

- ◯ Aldrig
- ◯ Sällan
- ◯ Ibland
- ◯ Ofta
- ◯ Alltid

När jag är i skolan känner jag mig fysiskt utmattad.

- ◯ Aldrig
- ◯ Sällan
- ◯ Ibland
- ◯ Ofta
- ◯ Alltid

Jag får kämpa för att uppbringa entusiasm för mitt skolarbete.

- ◯ Aldrig
- ◯ Sällan
- ◯ Ibland
- ◯ Ofta
- ◯ Alltid

Jag känner stark motvilja för mitt skolarbete.

- Aldrig
- Sällan
- Ibland
- Ofta
- Alltid

Jag känner mig cynisk inför vad mitt skolarbete har för betydelse för andra.

- Aldrig
- Sällan
- Ibland
- Ofta
- Alltid

Jag har svårt att vara fokuserad.

- Aldrig
- Sällan
- Ibland
- Ofta
- Alltid

Jag har svårt att koncentrera mig.

- Aldrig
- Sällan
- Ibland
- Ofta
- Alltid

Jag gör misstag eftersom jag tänker på annat.

- Aldrig
- Sällan
- Ibland
- Ofta
- Alltid

Jag känner mig oförmögen att kontrollera mina känslor.

- Aldrig
- Sällan
- Ibland
- Ofta
- Alltid

Jag känner inte igen mig själv i hur jag reagerar känslomässigt.

- Aldrig
- Sällan
- Ibland
- Ofta
- Alltid

Det händer att jag överreagerar i skolan utan att mena det.

- Aldrig
- Sällan
- Ibland
- Ofta
- Alltid

Din psykologiska trygghet

Besvara nedanstående påståenden utifrån den studentgrupp som du ingår i på skolan.

Om man gör ett misstag i min studentgrupp läggs det en oftast till last.

- ◯ Jag håller helt med
- ◯ Jag håller med
- ◯ Jag håller delvis med
- ◯ Jag håller varken med eller inte med
- ◯ Jag håller delvis inte med
- ◯ Jag håller inte med
- ◯ Jag håller verkligen inte med

Personer i min studentgrupp kan ta upp problem och svåra frågor.

- ◯ Jag håller helt med
- ◯ Jag håller med
- ◯ Jag håller delvis med
- ◯ Jag håller varken med eller inte med
- ◯ Jag håller delvis inte med
- ◯ Jag håller inte med
- ◯ Jag håller verkligen inte med

Personer i min studentgrupp stöter ibland ut andra för att de är annorlunda.

- ◯ Jag håller helt med
- ◯ Jag håller med
- ◯ Jag håller delvis med
- ◯ Jag håller varken med eller inte med
- ◯ Jag håller delvis inte med
- ◯ Jag håller inte med
- ◯ Jag håller verkligen inte med

Det känns tryggt att ta risker i min studentgrupp.

- Jag håller helt med
- Jag håller med
- Jag håller delvis med
- Jag håller varken med eller inte med
- Jag håller delvis inte med
- Jag håller inte med
- Jag håller verkligen inte med

Det är svårt att be andra i min studentgrupp om hjälp.

- Jag håller helt med
- Jag håller med
- Jag håller delvis med
- Jag håller varken med eller inte med
- Jag håller delvis inte med
- Jag håller inte med
- Jag håller verkligen inte med

Ingen i min studentgrupp skulle medvetet agera på ett sätt som undergräver mitt arbete.

- Jag håller helt med
- Jag håller med
- Jag håller delvis med
- Jag håller varken med eller inte med
- Jag håller delvis inte med
- Jag håller inte med
- Jag håller verkligen inte med

I min studentgrupp värdesätts och används mina unika förmågor och talanger.

- ◯ Jag håller helt med
- ◯ Jag håller med
- ◯ Jag håller delvis med
- ◯ Jag håller varken med eller inte med
- ◯ Jag håller delvis inte med
- ◯ Jag håller inte med
- ◯ Jag håller verkligen inte med
